# Immunosensors for Assay of Toxic Biological Warfare Agents

**DOI:** 10.3390/bios13030402

**Published:** 2023-03-20

**Authors:** Miroslav Pohanka

**Affiliations:** Faculty of Military Health Sciences, University of Defense, Trebesska 1575, CZ-50001 Hradec Kralove, Czech Republic; miroslav.pohanka@gmail.com or miroslav.pohanka@unob.cz

**Keywords:** Anthrax toxin, biosensor, botulinum toxin, cyanotoxin, immunoassay, ricin, Shiga toxin

## Abstract

An immunosensor for the assay of toxic biological warfare agents is a biosensor suitable for detecting hazardous substances such as aflatoxin, botulinum toxin, ricin, Shiga toxin, and others. The application of immunosensors is used in outdoor assays, point-of-care tests, as a spare method for more expensive devices, and even in the laboratory as a standard analytical method. Some immunosensors, such as automated flow-through analyzers or lateral flow tests, have been successfully commercialized as tools for toxins assay, but the research is ongoing. New devices are being developed, and the use of advanced materials and assay techniques make immunosensors highly competitive analytical devices in the field of toxic biological warfare agents assay. This review summarizes facts about current applications and new trends of immunosensors regarding recent papers in this area.

## 1. Introduction

Testing hazardous toxic materials is an important task in current analytical chemistry. Accurate and timely proof of hazardous materials in the environment or an organism is necessary for choosing the correct countermeasures or therapy. Various instrumental devices are available for the purpose, and accurate and sensitive assays of hazardous toxic materials are possible. Mass spectrometry, chromatography, electrophoresis, and immunochemical methods, such as enzyme-linked immunosorbent assay, can be standard analytical chemistry for toxins [1,2,3,4,5,6,7,8,9,10]. Although standard methods are available and fully applicable, they have disadvantages, such as the price of the device, cost per assay, and demands on staff and other laboratory equipment. Alternative methods are being sought, to serve in situations where standard methods are unsuitable. Simple devices usable in the field, small mobile laboratories, or by a sole investigator in terrain, or devices for point-of-care tests, could provide identification of toxins in sites where other methods are not convenient. 

Biosensors, chemosensors, aptasensors, and similar portable and low-cost analytical devices are generally suitable for use outside of laboratories. The concept of biosensors and biosensor-like devices brings an alternative to standard methods because the application of new materials and measurement procedures makes them sensitive up to the level of these standard methods [11,12]. They maintain the concept of simple portable devices that can even be integrated as wearable electronics in the future. 

Toxins with military relevance represent a group of harmful substances with serious pathological impacts on the human organism. The test of such toxins with small portable devices is highly desired. It can protect endangered persons, help choose proper therapy, and diagnose the true causative agent of poisoning. Biosensors with bound antibodies, immunosensors, are reviewed here. Recent discoveries are introduced, and the significance of immunosensors is discussed. 

## 2. Toxins as a Part of Biological Warfare Agents

Nuclear, radiological, chemical, and biological weapons of mass destruction exist. They are a threat when used by a state in war, by an organization, or by an individual perpetrator in a terrorist attack [13,14]. All types of mass destruction weapons are regulated by international treaties, and there is an effort to ban or at least restrict their possession. *The Treaty on the Non-Proliferation of Nuclear Weapons* from 1968, effective as of 1970, is the main international regulation for the first group of weapons of mass destruction. Most countries declared for abandoning nuclear weapons, except for Great Power states. Chemical and biological warfare agents are partially regulated worldwide per the so-called Geneva protocols of 1925. The *Protocol for the Prohibition of the Use in War of Asphyxiating, Poisonous or other Gases, and of Bacteriological Methods of Warfare*, however, was minimally effective. It did not force the signatories to stop arming themselves with these weapons; therefore, further treaties followed in the next decades. The *Convention on the Prohibition of the Development, Production and Stockpiling of Bacteriological (Biological) and Toxin Weapons and on their Destruction* is the treaty regulating biological warfare. It was signed by most countries in the world in 1972 and entered into force in 1975. Chemical warfare agents have become fully banned internationally, the last of the mass destruction weapons. The *Convention on the Prohibition of the Development, Production, Stockpiling and Use of Chemical Weapons and on their Destruction* was signed in 1993 and entered into force in 1997.

Despite extensive regulation of mass destruction weapon manufacturing, stockpiling, and use, their relevance and threat are still significant. The proliferation of such means of combat or terror can occur under certain circumstances, and active countermeasures still exist to protect against such threats [15,16,17,18,19]. 

Toxins are poisons of natural origin. They can be simple organic compounds and/or highly structurally arranged macromolecules. Anatoxin-a, with a molecular mass of 165 Da, and botulinum toxin, with a molecular mass of 150 kDa, can be mentioned as two toxic biological warfare agents of completely different sizes. Several toxins are considered biological warfare agents, and often the toxin itself and the producing microorganism are seen as a biological threat [20]. Functional subunits derived from the sizable toxins also have the status of biological warfare agents. 

Many toxic substances could be considered biological warfare agents; however, only a limited number have this status in practice. For instance, The Australia Group (Australia Group Secretariat, RG Casey Building, John McEwen Crescent, Barton Act 0221) coordinating 42 countries, plus the European Union, has a list of human and animal pathogens and toxins for export control. A total of 18 structurally close groups of toxins are on the list. Abrin (a protein toxalbumin from the plant *Abrus pulchellus*) [21], aflatoxins (a low molecular weight mycotoxins from molds *Aspergillus* species) [22], botulinum toxins (all variants, protein toxins from the bacterium *Clostridium botulinum*) [23], cholera toxin (a protein toxin from the bacterium *Vibrio cholerae*) [24], Clostridium perfringens toxins (protein α, β1, β2, ε, ι toxins from bacterium *Clostridium perfringens*) [25], conotoxins (a group of toxic peptides from marine cone snail, genus *Conus*) [26], diacetoxyscirpenol (a low molecular weight mycotoxin from a group of trichothecenes and produced by the Fusarium fungi) [27], HT-2 toxin (a trichothecene mycotoxin produced by various fungi mainly of *Fusarium* species) [28], microcystins (cyanotoxins, a group of organic compounds produced by cyanobacteria) [29], modeccin (a glycoprotein from plant *Adenia digitata*), ricin (a carbohydrate binding protein from plant *Ricinus communis*) [30], saxitoxin (a cyanotoxin from various cyanobacteria, organic compound) [31], Shiga toxins (including Shiga-like toxins, verotoxins and verocytotoxins, a group of protein toxins from *Shigella dysenteriae* and some serotypes of *Escherichia coli*) [32], Staphylococcus aureus enterotoxins (including hemolysin α-toxin and toxic shock syndrome toxin, a group of protein toxins from bacterium *Staphylococcus aureus*), T-2 toxin (a trichothecene mycotoxin produced by various fungi mainly of *Fusarium* species) [33], tetrodotoxin (a neurotoxin organic substance produced by bacteria like *Pseudoalteromonas*, *Pseudomonas*, and *Vibrio*, it can be transmitted to other water organisms) [34], viscumin (viscumin albumin lectin 1, toxic lectins from mistletoe plant *Viscum album*) [35], and volkensin (a toxic glycoprotein from *Adenia volkensii* plant) [36] are regulated substances according to the Australia Group. The mentioned toxins are given in Table 1.

The Center for Disease Control and Prevention (1600 Clifton Road, Atlanta, GA 30329-4027 USA) distinguishes three basic types of biological warfare agents labeled A, B, and C [37,38]. Group A contains the most dangerous biological warfare agents. Groups B and C are less important as the agents are less dangerous. Serious pathogens, such as *Bacillus anthracis*, *Francisella tularensis*, *Yersinia pestis,* and *Variola major* belong to group A. *Clostridium botulinum* toxin (Botulinum toxin) also belongs to the upper-priority group A as a representative of toxic substances. 

## 3. Biosensors for the Toxic Biological Warfare Agents Assay

**Figure 1:**
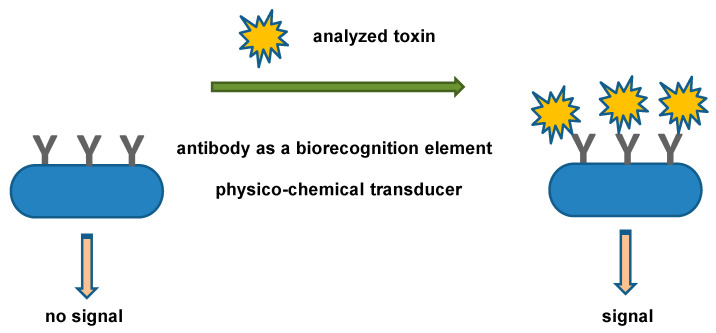
General principle of an immunosensor for toxin assay.

Biosensors are analytical devices that combine a physicochemical transducer and a biorecognition element. While the physicochemical transducers work as a physical sensor, the biorecognition element is responsible for specificity, but it can also initiate chemical or physical processes detectable by the physico-chemical transducer. Biosensor analytical devices have progressed from simple detectors containing crude enzymes, such as glucose oxidase, to complex systems where purposely prepared biological origin molecules, nanomaterials, and other advanced techniques are used [39,40,41,42]. Immunosensors are a variant of a biosensor where an antibody plays the role of a biorecognition element, and an antigen is an analyte [43,44,45,46,47]. Conception in which an immunosensor containing an antibody is detected by an antigen is possible as well [48]. Toxins assay by an immunosensor can work on a direct interaction between immobilized antibodies specific to the toxin and the toxin itself presented in the sample. More complicated assay formats also exist, and sandwich immunocomplexes, competitive immunoassays, formation of complexes with nanoparticles, and other arrangements are known, as described in the chapter devoted to the specific examples. A general principle of an immunosensor for toxin assay is shown in Figure 1. 

Biological warfare agents, including toxins, can be analyzed by a wide number of biosensors, as seen in the examples in the following text. The use of antibodies as a biorecognition element is quite common for a biological warfare agent assay. An electrochemical paper immunosensor for a *B. anthracis* assay is an example [49]. Antibodies react with a target molecule, called an antigen, and specifically recognize a site on the antigen called a paratope. The use of antibodies in various analyses has a long tradition, and specific antibodies can be attained in the market. On the other hand, antibodies are sizable molecules, and their production requires the use of animals (polyclonal antibodies) or biotechnology (monoclonal and recombinant antibodies). This means that the production of antibodies is not easily reproducible and can also require a high initial investment in material and work. 

Aptamers are another recognition element representing an artificial molecule based on polynucleotides, polydeoxynucleotides, or peptide-binding [50,51]. The use of aptamers for analyses became quite common, and the term aptasensor can be found in the current literature. Using an aptasensor for biological warfare agents is possible, and the application of bacillus anthracis is an example [52,53]. Aptamers exert affinity to the target molecule as antibodies do. Because aptamers are artificial biomolecules, they can be produced by typical chemical technologies, and thus the product can be more attractive to some manufacturers. On the other hand, aptamers can have problems with specificity and affinity concerning the target structures, though their production technology is proven, and some aptamers have good specifications. 

Molecularly imprinted polymers are another affinity material manufactured via chemical processes. Molecularly imprinted polymers can serve in the same way as a biorecognition element and gain specificity to the sensor device [54,55,56]. Molecularly imprinted polymers could be mass-produced by the chemical industry, and any structure can be imprinted in theory. There are, however, some shortcomings that should be taken into account. The specificity of the imprints can be limited. The affinity of the surface to the target molecule is based on the shape and molecular interactions, which are not guaranteed when a homogenous membrane is used, and testing small molecules with defined physical and chemical specifications is an easier task for sensors with molecularly imprinted polymers. Experiences with molecularly imprinted polymers for the preparation of sensors include the *Helicobacter pylori* virulence factor assay [57], specific extraction of aflatoxins by molecularly imprinted polymers [58], and the human immunodeficiency virus drug assay by Tenofovir [59]. The use of molecularly imprinted polymer will gain more applications when new materials are developed as a platform for in situ membrane manufacturing. 

Biological warfare agents can be analyzed by recognizing specific genes or sequences of their genetic information and using genetic probes, specific sequences of genetic information, etc. These devices proved their functionality in more applications, such as the Ebola virus assay [60,61], *F. tularensis* [62], *F. tularensis*, *Y. pestis*, *B. anthracis*, variola virus, Rift Valley fever virus Ebola virus, Sudan virus, and Marburg virus [63], variola major [64], *B. anthracis* [65,66], and Shiga toxin-producing *E. coli* [67]. Identifying biological warfare agents by detecting their genetic sequences is typically a sensitive and selective approach. These assays also have some disadvantages. First, genetic information is enclosed within cells or viral particles, and only rarely can the genetic information be attained directly. This may cause complications and will probably make it necessary to pretreat samples. Another disadvantage is that toxins cannot be assayed directly by genetic tests. Only a microorganism that produces the toxins can be analyzed. 

Biosensors, including immunosensors, are a group of portable analytical devices. Some highly complex biosensors are not suitable for use outside of laboratories, but most of the newly developed biosensors are miniaturized instruments suitable for field tests. The immunosensor for toxic biological warfare agents plays a role in fast identification in order to choose proper countermeasures. The immunosensor should be a single step or based on a limited number of steps, with a minimal requirement of sample pretreatment and personnel operating the device. It is not expected that the immunosensor will replace standard laboratory analytical methods, such as chromatography or mass spectrometry. The standard methods should verify results from the immunosensor when the situation allows. The role of an immunosensor in the toxic biological warfare agent assay can be compared with point-of-care tests based on lateral flow immunochromatography that were found to be useful during the Coronavirus 2019 pandmic, and that were taken as a less expensive, accessible, but less accurate diagnostic alternative to the standard polymerase chain reaction assays [68,69,70,71,72,73].

Biosensors also have shortcomings that should be considered when a new analytical device containing a biosensor is constructed. Compared to the universal standard analytical devices, biosensors are suitable for assaying a specific analyte or a group of defined analytes. The specificity depends on the type of biorecognition element or manufactured molecule. The recognition antibody has to be replaced in the case of an immunosensor for a toxic biological warfare agents assay. This replacement is quite elaborate and cannot be done by a user. Therefore, immunosensors are not universal devices but analytical tools for specific tasks. 

## 4. Commercial Immunosensors for Toxic Biological Warfare Agents

The research on immunosensors for toxic biological warfare agents is ongoing, and many interesting applications have already been commercialized. The already commercialized devices are outcomes of older research, and they have an actual use for safety purposes. On the other hand, the actual research outcomes are not involved in their construction. Both expensive automatically working analytical devices and cheap disposable detectors can be mentioned as successful adaptations of an immunosensor for the assay of biological warfare agents, including toxins. 

**Figure 2:**
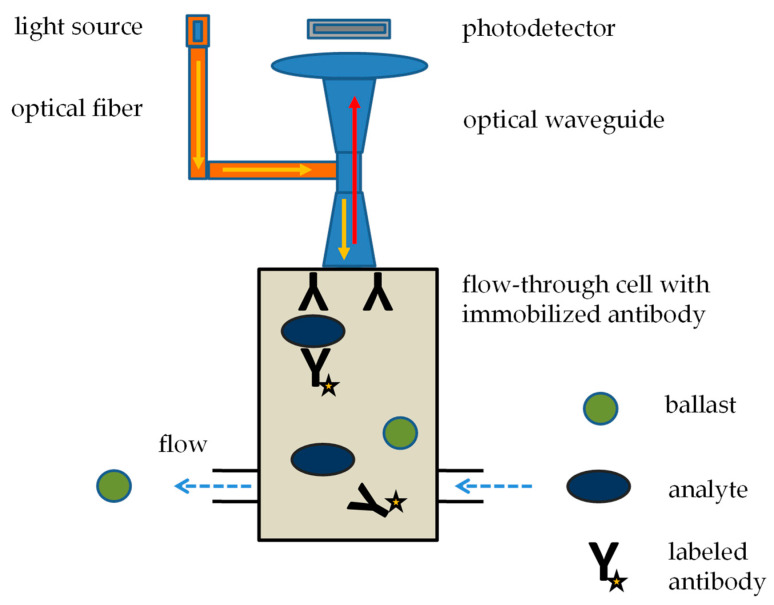
Principle of Raptor analytical device based on a fluorescence immunoassay. Blue arrow: liquid flow, yellow arrow: exciting light, red arrow: emitted light.

The analyzer Raptor by Research International (Monroe, WA, USA) is an automatic, portable fluorometric assay system for monitoring up to four toxins, viruses, bacteria, spores, fungi, and other diverse targets, and it can be designated an immunosensor. It is a battery-powered portable device of 28.0 × 17.3 × 20.5 cm and 6.45 kg and is suitable for indoor and outdoor applications. It works on the principle of fluorescence immunoassay, which takes place in four independent channels, meaning that up to four biological warfare agents can be analyzed simultaneously. One assay takes 15 min to complete. All steps are automated, and flow forced by a peristaltic pump is responsible for the delivery of samples and the solutions of monoclonal antibodies with bound fluorophore labels to a chamber where another antibody has already been immobilized. Optical fibers excite the fluorophore, and an optical waveguide detects fluorescence when an immunocomplex with the analyte is formed in the flow-through cell. The principle of the Raptor function is depicted in Figure 2. The Raptor device can analyze a wide group of biological warfare agents. The exact type of agent depends on the regencies used. Toxic biological warfare agents can be proven with quite low detection limits: up to 0.1 ng/mL for staphylococcal enterotoxin B, 5 ng/mL for ricin, and up to 1 ng/mL for botulinum toxin [74,75,76,77,78,79,80]. 

Fluorescence measurement also uses another immunosensor for a biological warfare agents assay: Biosensor 220R by MSA (Pittsburgh, PA, USA). This immunosensor works automatically and uses magnetic microspheres with specific antibodies and a fluorescent tag with specific antibodies [81]. A complex is formed when an analyte is presented in a sample, the complex is held in a flow through a magnetic cell and washed, and its fluorescence is measured. The manufacturer does not disclose detailed information about the magnetic particles and antibodies. The whole device is suitable for indoor and outdoor performance. It is battery-powered, 27 × 25 × 14 cm in size, and weighs 2.7 kg. The manufacturer claims a sensitivity for ricin and staphylococcal enterotoxin B < 1 ng for an assay lasting 5 min.

**Figure 3:**
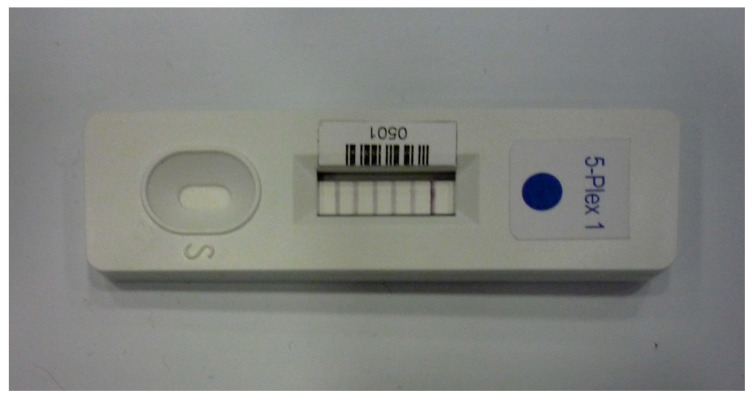
A commercial lateral flow test for contemporary assay of biological warfare agents.

Lateral flow tests, also known as lateral flow immunoassays, are an analytical tool for the semiquantitative analysis of various chemicals, drugs, semiquantitative substances, biochemical and immunochemical markers, and microorganisms [82,83,84,85,86,87,88]. Toxic biological warfare agents can also be analyzed by lateral flow tests, and some manufacturers offer these immunoassay devices specific to toxins of security interest. Manufacturer Advnt Biotechnologies (Phoenix, AZ, USA) produces lateral flow tests for various biological warfare agents. There are tests for a single agent or for up to five agents analyzed in one assay. The tests for a single agent are named BADD (Biowarfare Agent Detection Devices); the tests for five simultaneous agents are called the Pro Strips Rapid Screening System. Other analytical specifications are the same for both tests. The detection limit for ricin and staphylococcal enterotoxin B is 10 ng/mL, the botulinum toxin variant A has a detection limit of 33 ng/mL, and the botulinum toxin variant B has a detection limit of 500 ng/mL for an assay that requires a sample size of 0.2 mL and a time of 3 min. The practical use of these strips was described in the papers cited for the assay of ricin [89] and the A variant of botulinum toxin [90]. Alexeter Technologies manufactures (Wheeling, IL, USA) similar lateral flow tests under the trade name BioDetect (test of a single biological warfare agent), RAID 5 (up to five contemporary assayed biological warfare agents), RAID 8 (up to eight contemporary assayed biological warfare agents), and RAID 10 (up to 10 contemporary assayed biological warfare agents). Ricin, staphylococcal enterotoxin B, and botulinum toxin are covered by these tests, but the manufacturer does not offer an assay for other toxic biological warfare agents. The assay takes 15 min to complete, though other analytical specifications are not disclosed by the manufacturer. Practical testing for ricin was described by Slotved et al. [89]. Lateral flow tests are also produced by other manufacturers. ANP Technologies (Newark, DE, USA) produce lateral flow tests for biological warfare agents and infectious microorganisms. Botulinum toxin A, ricin, and staphylococcal enterotoxin B tests are offered as tools for toxin assay. The manufacturer provides the tests for single target and multiplex assays suitable for the contemporary detection of two, four, five, and ten biological warfare agents. An example of a multiplexed lateral flow test is depicted in Figure 3. 

The assay by lateral flow test can be further improved by using a digital reader to measure the coloration of lines, and automatizes manipulation with samples. The device BioHawk LF by Research International is an example. It can even collect samples from aerosols via an external wetted wall cyclone, and perform automated detection of biological warfare agents and their identification in a total elapsed time of 10–25 min. The device is suitable for outdoor use, and its small size of 47.0 × 24.8 × 36.5 cm with a weight of 13 kg makes it single-person portable. The commercially available immunosensors for the assay of toxic biological warfare agents are summarized in Table 2. 

## 5. Progress on Immunosensors for Toxic Biological Warfare Agents Assay

Research on a new immunosensor for the assay of toxic biological warfare agents brings improvements, making devices more competitive to standard methods. New materials typically improve expected specifications, such as decreasing limits of detection and sample volume on one side and making the assay simple on the other. Miniaturization additionally leads to savings on raw materials and production costs.

An immunosensor that works on the principle of the Raman scattering-lateral flow immunoassay was developed by Jia et al. [91]. Composite gold—silicon oxide nanoparticles were chosen for the assay as fluorescent labels. Variants of the immunosensor for the ricin, botulinum toxin, and staphylococcal enterotoxin B assay were developed. The toxins were analyzed with a detection limit of 0.1 ng/mL for ricin and botulinum toxin A, of 0.05 ng/mL for staphylococcal enterotoxin B, and the time per single measurement was 15 min. A voltametric immunosensor was developed to detect vacuolating cytotoxin A from *Helicobacter pylori* [92]. Although this toxin is not listed among biological warfare agents, the assay provides promising results and can be easily adapted for other bacterial toxins. The authors prepared a graphitic carbon nitride/zinc oxide nanocomposite electrochemically deposited on gold electrodes, further immobilized antibodies via carbodiimide and N-hydroxysuccinimide, and vacuolating cytotoxin A was detected by voltammetry. The detection limit for the assay was equal to 0.1 ng/mL for vacuolating cytotoxin A with a linear range of calibration between 0.1 and 12.8 ng/mL and a time per test of 10–15 min. An electrochemiluminescence immunosensor for a ricin assay was developed on a platform of screen-printed electrodes [93]. The immunosensor contained magnetic beads with antibodies specific for ricin immobilized through streptavidin-biotin. A sandwich was formed in the presence of ricin with CdSe/ZnS quantum dots, the immunocomplex formed on the magnetic beads was magnetically separated, and electrochemiluminescence was measured. The immunosensor had a detection limit of 5.5 pg/mL and a linear assay range of 0.01–100 ng/mL. Magnetic beads were also used in the work by Atanasova and colleagues concerning the detection of aflatoxin M1 [94]. The magnetic nanoparticle-based fluorescent immunoassay provided a limit of detection for aflatoxin M1 2.9 pg/mL and a linear calibration range of 3.0 to 100 pg/mL. An immunosensor for aflatoxins was also developed in the work of Peltomaa et al. [95]. They developed a non-competitive immunoassay in which a primary anti-aflatoxin antibody was bound via streptavidin to magnetic beads, and an immunocomplex was formed in the presence of aflatoxin B1 with a secondary Eu-labeled antibody. Fluorescence was measured after the magnetic separation. The assay had a detection limit of 70 pg/mL for an assay lasting 15 min.

Botulinum toxin A was measured by an immunosensor, in which specific antibodies were attached to gold nanoparticles, a sandwich immunocomplex was formed with botulinum toxin and antibodies on fluorescent probe particles, and diffusivity was measured [96]. The assay had a detection limit of 10 pg/mL for a measurement time of 2 min, and botulinum toxin A was measured in a calibration range of 0.01–500 ng/mL. In another work, the simultaneous detection of botulinum toxins A and E was performed by a voltametric assay [97]. The immunosensor comprised magnetic core/metal-organic framework nanoparticles covered with antibodies specific to botulinum toxins and monoclonal antibodies labeled with polystyrene@polydopamine/cadmium and silver. The assay had a dynamic range of 0.1–1000 pg/mL and a limit of detection of 0.04 pg/mL for botulinum toxin A, and a dynamic range of 0.5–1000 pg/mL and a limit of detection of 0.16 pg/mL for botulinum toxin E. 

The botulinum toxin assay was also developed in the work of Kumar et al. [98]. They chose the toxoid form of botulinum toxin types C and D for their analysis, and the porous silicon Fabry-Perot interferometer as a platform for a competitive immunoassay. It was covered with a gelatin membrane and botulinum toxoid. Primary antibodies specific for toxoid and secondary antibodies labeled with horse radish peroxidase were used, and peroxidase-catalyzed oxidation of 4-chloro-1-naphthol using hydrogen peroxide created insoluble products. The botulinum toxin in a sample was completed with the immobilized toxoid for the antibodies applied. Reflectivity spectra were collected, and calibration was performed. The assay had a linear response of 10 pg/mL to 10 ng/mL and a limit of detection of 4.8 pg/mL for an assay occurring in nearly real-time. 

The immunosensor for toxins can also use complex and more expensive platforms to achieve outstanding specifications. Shiga toxins were, for instance, analyzed with surface plasmon resonance imaging [99]. This immunosensor contained immobilized immunoglobulin G on 50 nm gold film and proved Shiga toxoid Stx1 in a label-free mode with a detection limit of 50 ng/mL in an assay lasting 20 min. The signal can be further improved by applying gold nanoparticles covered with anti-Shiga toxin antibodies. The sensitivity of the assay improves when the immunosandwich forms, and the limit of detection is around 1 pg/mL. Surface plasmon resonance was used to detect ricin and abrin in another article [100]. A sandwich immunocomplex comprised of a protein G, a magnetic bead with an antibody, analyte, and secondary antibody, was formed and placed at the site of the proper sensor chip. The assay contained a magnetic separation step that enriched the analyte and improved sensitivity. The limit of detection for abrin and ricin assay was equal to 0.6 ng/mL. Immunocomplex formation on the surface plasmon resonance chip was also used in work by Stern et al. [101]. The authors co-immobilized antibodies against ricin, and agglutinins were assayed in the first step. Adding an antibody specific for ricin formed a sandwich immunocomplex, and the level of ricin could be differentiated from the level of agglutinin. The detection limit was equal to 3 ng/mL for ricin and 6 ng/mL for agglutinin in an assay providing the assay results in real-time. The total analysis time, including sample processing, was less than 30 min. The newly developed immunosensors for the toxic biological warfare agent assay are summarized in Table 3.

Introducing new immunosensors into practice is not an easy task. It requires not only assembling the particular parts but also using original nanomaterials and antibodies, and their production is a condition for getting an immunosensor into the market. Generally, producing biosensors and immunosensors has a great practical perspective, and their use by various consumers is expected [102,103]. Immunosensors for toxic biological warfare agents assays are devices designed for the military, police, or other organizations. The introduction of immunosensors to these consumers will highly depend on governmental support or acquisitions. The fact that one immunosensor typically detects only one type of toxic biological warfare agent is a disadvantage. Militaries tend to require a single analyzer for a wide number of analytes, and that the analyses are performed by trained staff for whom education in analytical chemistry, bioanalytical chemistry, or similar disciplines is necessary. There can also be problems with the manufacturing processes in which new materials are used, and shortcomings in quality or reproducibility can occur. The limitations mentioned here should be considered when the introduction of an immunosensor is planned. On the other hand, the benefits of small, portable, and cheap analytical devices for security practices are undeniable. The practical spread of the immunosensor for toxic biological warfare agents will depend on the verification of their potential by military specialists. If the first of the new types of immunosensors are at least partially successful, further propagation of them for toxic biological warfare agents can be expected. 

## 6. Conclusions

Toxins represent a substantial risk to human health; they can be present in the environment, food, and drugs or accompany infectious diseases. They are also a threat that can be misused for military or terrorist activities. Early detection is a necessity for helping to decide what countermeasures or therapies should be chosen. Although current analytical techniques are accurate and reliable, early test detection for outdoor measurement or point-of-care diagnosis is extremely helpful. Immunosensors can provide a highly sensitive assay and the possibility to perform testing outside of standard laboratories. Recent discoveries and the implementation of new materials make immunosensors highly sensitive and capable of detecting toxins in very low concentrations. At the same time, these devices are typically inexpensive, small, are readily integrated into portable or even wearable electronics, and perform point-of-care tests. The currently commercialized immunosensors are fully applicable. The newly developed ones will further improve possibilities for toxin assay. 

## Figures and Tables

**Table 1:** Toxins with relevance as biological warfare agents.

Toxin	Type of Chemical Substance	Producing Organism	References
abrin	protein toxalbumin	plant *Abrus pulchellus*	[21]
aflatoxin	low molecular weight mycotoxins	molds *Aspergillus*	[22]
botulinum toxins	protein toxins	bacterium *Clostridium botulinum*	[23]
cholera toxin	protein toxins	bacterium *Vibrio cholerae*	[24]
Clostridium perfringens toxins	protein α, β1, β2, ε, ι toxins	bacterium *Clostridium perfringens*	[25]
conotoxins	neurotoxic peptides	marine cone snail, genus *Conus*	[26]
diacetoxyscirpenol	a low molecular weight mycotoxin from a group of trichothecenes	produced by fungi *Fusarium*	[27]
HT-2 toxin	a trichothecene mycotoxin	various fungi, mainly *Fusarium* species	[28]
microcystins	cyanotoxins, a group of organic compounds	various cyanobacteria	[29]
modeccin	a glycoprotein	plant *Adenia digitata*	
ricin	a carbohydrate-binding protein	plant *Ricinus communis*	[30]
saxitoxin	a cyanotoxin, organic compound	various cyanobacteria	[31]
Shiga toxins	a group of protein toxins	*Shigella dysenteriae* and some serotypes of *Escherichia coli*	[32]
T-2 toxin	a trichothecene mycotoxin	produced by various fungi, mainly *Fusarium* species	[33]
tetrodotoxin	an organic neurotoxic substance	bacteria like *Pseudoalteromonas*, *Pseudomonas*, and *Vibrio*, it can be transmitted to other water organisms	[34]
viscumin	toxic protein lectins	mistletoe plant *Viscum album*	[35]
volkensin	a toxic glycoprotein	*Adenia volkensii* plant	[36]

**Table 2:** Commercially available immunosensors for assay of toxic biological warfare agents.

Name of Device	Manufacturer	Type of Immunosensor or Assay	Analytical Specifications	References
Raptor	Research International (Monroe, WA, USA)	automatic flow through fluorescence immunoassay	limits of detection up to 0.1 ng/mL for staphylococcal enterotoxin B, 5 ng/mL for ricin, and up to 1 ng/mL for botulinum toxin, assay time 15 min	[74,75,76,77,78,79,80]
Biosensor 220R	MSA (Pittsburgh, PA, USA)	fluorescence immunoassay based on magnetic separation	sensitivity for ricin and staphylococcal enterotoxin B < 1 ng, assay time 5 min	[81]
BADD and Pro Strips-Rapid Screening System	Advent Biotechnologies	lateral flow test	limit of detection for ricin and staphylococcal enterotoxin B is 10 ng/mL, botulinum toxin variant A 33 ng/mL, botulinum toxin variant B 500 ng/mL, sample sized 0.2 mL, assay time 3 min, contemporary analyzed biological warfare agents: 1 or 5	[89,90]
BioDetec, RAID 5, RAID 8, RAID 10	Alexeter Technologies	lateral flow test	assay time 15 min, contemporary analyzed biological warfare agents: 1, 5, 8 or 10	[89]

**Table 3:** New immunosensors for toxic biological warfare agents assay.

Type of Assay	Toxins	Analytical Specifications	References
Raman scattering-lateral flow immunoassay	ricin, botulinum toxin, and staphylococcal enterotoxin B	limit of detection 0.1 ng/mL for ricin and botulinum toxin A, and 0.05 ng/mL for staphylococcal enterotoxin B, assay time 15 min	[91]
voltametric immunoassay	vacuolating cytotoxin A from *Helicobacter pylori*	limit of detection 0.1 ng/mL, linear range of calibration between 0.1 and 12.8 ng/mL, assay time 10–15 min	[92]
electrochemiluminescence immunosensor with magnetic separation of immunocomplex on magnetic beads	ricin	limit of detection 5.5 pg/mL, linear assay range 0.01–100 ng/ml	[93]
magnetic nanoparticle-based fluorescent immunoassay	aflatoxin M1	limit of detection 2.9 pg/mL, linear calibration range 3.0–100 pg/ml	[94]
non-competitive immunoassay, primary anti-aflatoxin antibody bound via streptavidin on magnetic beads, an immunocomplex is formed in the presence of aflatoxin B1 with a secondary Eu-labelled antibody	aflatoxin B1	limit of detection 70 pg/mL, assay time 15 min	[95]
diffusivity measurement of sandwich immunocomplexes comprised of gold nanoparticles with antibodies, analyte, and antibodies on fluorescent probe particles	botulinum toxin	limit of detection 10 pg/mL, calibration range 0.01–500 ng/mL, assay time 2 min	[96]
voltametric immunosensor containing magnetic particles with antibodies forming a sandwich with analyte and other antibodies labeled with Ag or Cd nanoparticles	botulinum toxin A and E	dynamic range 0.1–1000 pg/mL and limit of detection 0.04 pg/mL (botulinum toxin A); dynamic range 0.5–1000 pg/mL and limit of detection 0.16 pg/mL (botulinum toxin E)	[97]
Fabry-Perot interferometric competitive immunoassay using primary and peroxidase-labeled secondary antibody, precipitation of 4-chloro-1-naphthol by peroxidase was responsible for the detected signal	toxoid form of botulinum toxin type C and D	linear response 10 pg/mL to 10 ng/mL, limit of detection 4.8 pg/mL, assay going in nearly real time	[98]
surface plasmon resonance imaging, antibody bound on gold film, signal improved by adding of gold nanoparticles with immobilized antibodies	Shiga toxin—tested on toxoid	limit of detection 50 ng/mL for label-free assay, 1 pg/mL when gold-immuno-nanoparticles are applied, assay time 20 min	[99]
surface plasmon resonance combined with magnetic separation	ricin and abrin	limit of detection 0.6 ng/ml	[100]
surface plasmon resonance with antibodies immobilized on chip and secondary antibody used for specific ricin assay and signal improvement	ricin, agglutinin	3 ng/mL for ricin, 6 ng/mL for agglutinin, assay time including sample processing 30 min	[101]

## Data Availability

All data are presented in this work.

## References

[1] Lee C.W., Su H., Shiea J. (2022). Potential applications and challenges of novel ambient ionization mass spectrometric techniques in the emergency care for acute poisoning. Trac-Trends Anal. Chem..

[2] De Girolamo A., Lippolis V., Pascale M. (2022). Overview of recent liquid chromatography mass spectrometry-based methods for natural toxins detection in food products. Toxins.

[3] Su H., Huang M.Z., Shiea J.T., Lee C.W. (2023). Thermal desorption ambient ionization mass spectrometry for emergency toxicology. Mass Spectrom. Rev..

[4] Tittlemier S.A., Cramer B., Dall’Asta C., Iha M.H., Lattanzio V.M.T., Maragos C., Solfrizzo M., Stranska M., Stroka J., Sumarah M. (2020). Developments in mycotoxin analysis: An update for 2018-19. World Mycotoxin J..

[5] Liew W.P.P., Sabran M.R. (2022). Recent advances in immunoassay-based mycotoxin analysis and toxicogenomic technologies. J. Food Drug Anal..

[6] Amin R., Alam F., Dey B.K., Mandhadi J.R., Bin Emran T., Khandaker M.U., Safi S.Z. (2022). Multidimensional chromatography and its applications in food products, biological samples and toxin products: A comprehensive review. Separations.

[7] Valdes A., Alvarez-Rivera G., Socas-Rodriguez B., Herrero M., Cifuentes A. (2022). Capillary electromigration methods for food analysis and foodomics: Advances and applications in the period february 2019-february 2021. Electrophoresis.

[8] Bouteiller P., Lance E., Guerin T., Bire R. (2022). Analysis of total-forms of cyanotoxins microcystins in biological matrices: A methodological review. Toxins.

[9] Hempel B.F., Damm M., Petras D., Kazandjian T.D., Szentiks C.A., Fritsch G., Nebrich G., Casewell N.R., Klein O., Sussmuth R.D. (2023). Spatial venomics-cobra venom system reveals spatial differentiation of snake toxins by mass spectrometry imaging. J. Proteome Res..

[10] Zhao Z.Y., Hengchao E., Tian E.J., Fan T.T., Yang X.L., Li X.B., Zhang Y.M., Li X.J., Chen A.L., Zhou C.Y. (2023). Structural annotation and discovery of toxic cyclopeptides and their analogues in lethal mushroom amanita and lepiota species using uplc-hrms and molecular networking strategy. Food Control.

[11] Zhou J.J., Lv X.Q., Jia J.L., Din Z.U., Cai S.Q., He J.L., Xie F., Cai J. (2022). Nanomaterials-based electrochemiluminescence biosensors for food analysis: Recent developments and future directions. Biosensors.

[12] Pohanka M. (2022). Progress in biosensors for the point-of-care diagnosis of COVID-19. Sensors.

[13] Hignett S., Hancox G., Otter M.E. (2019). Chemical, biological, radiological, nuclear and explosive (cbrne) events systematic literature review of evacuation, triage and decontamination for vulnerable people. Int. J. Emerg. Serv..

[14] Razak S., Hignett S., Barnes J. (2018). Emergency department response to chemical, biological, radiological, nuclear, and explosive events: A systematic review. Prehospital Disaster Med..

[15] Mueller J., Mueller K. (2000). The methodology of mass destruction: Assessing threats in the new world order. J. Strateg. Stud..

[16] Peintner L., Wagner E., Shin A., Tukhanova N., Turebekov N., Abdiyeva K., Spaiser O., Serebrennikova Y., Tintrup E., Dmitrovskiy A. (2021). Eight years of collaboration on biosafety and biosecurity issues between kazakhstan and germany as part of the german biosecurity programme and the g7 global partnership against the spread of weapons and materials of mass destruction. Front. Public Health.

[17] Graham A.T. (2021). The nuclear non-proliferation treaty: Delayed review—Issues old and new. J. Peace Nucl. Disarm..

[18] Vogel H. (2007). Weapons of mass destruction, wmd. Eur. J. Radiol..

[19] Priego A. (2014). Mass-destruction weapons proliferation in the national security strategy 2013. Rev. UNISCI.

[20] Janik E., Ceremuga M., Saluk-Bijak J., Bijak M. (2019). Biological toxins as the potential tools for bioterrorism. Int. J. Mol. Sci..

[21] Olsnes S. (2004). The history of ricin, abrin and related toxins. Toxicon.

[22] Ahmad M.M., Qamar F., Saifi M., Abdin M.Z. (2022). Natural inhibitors: A sustainable way to combat aflatoxins. Front. Microbiol..

[23] Pohanka M. (2020). Botulinum toxin as a biological warfare agent: Poisoning, diagnosis and countermeasures. Mini-Rev. Med. Chem..

[24] Rostami A., Zadeh F.A., Ebrahimzadeh F., Jafari-Sales A., Gholami S. (2022). Globally vibrio cholera antibiotics resistance to rna and DNA effective antibiotics: A systematic review and meta-analysis. Microb. Pathog..

[25] Morris W.E., Fernandez-Miyakawa M.E. (2009). Toxins of clostridium perfringens. Rev. Argent. Microbiol..

[26] Dao F.Y., Yang H., Su Z.D., Yang W.R.T., Wu Y., Ding H., Chen W., Tang H., Lin H. (2017). Recent advances in conotoxin classification by using machine learning methods. Molecules.

[27] Schollenberger M., Drochner W., Muller H.M. (2007). Fusarium toxins of the scirpentriol subgroup: A review. Mycopathologia.

[28] Imathiu S.M., Edwards S.G., Ray R.V., Back M.A. (2013). Fusarium langsethiae—A ht-2 and t-2 toxins producer that needs more attention. J. Phytopathol..

[29] Welten R.D., Meneely J.P., Elliott C.T. (2020). A comparative review of the effect of microcystin-lr on the proteome. Expo. Health.

[30] Kozlov Y.V., Sudarkina O.Y., Kurmanova A.G. (2006). Ribosome-inactivating lectins of plants. Mol. Biol..

[31] Akbar M.A., Yusof N.Y.M., Tahir N.I., Ahmad A., Usup G., Sahrani F.K., Bunawan H. (2020). Biosynthesis of saxitoxin in marine dinoflagellates: An omics perspective. Mar. Drugs.

[32] Bergan J., Lingelem A.B.D., Simm R., Skotland T., Sandvig K. (2012). Shiga toxins. Toxicon.

[33] Leal M., de Mejia E.G. (1997). Review: Toxicological and nutritional implications of t-2 toxin. Food Sci. Technol. Int..

[34] Makarova M., Rycek L., Hajicek J., Baidilov D., Hudlicky T. (2019). Tetrodotoxin: History, biology, and synthesis. Angew. Chem.-Int. Edit..

[35] Maltseva D.V., Gerasimov V.M., Sakharov D.A., Shkurnikov M.Y. (2017). Target cell glycosylation determines the biodistribution of plant lectin viscumin. Bull. Exp. Biol. Med..

[36] Battelli M.G., Musiani S., Buonamici L., Santi S., Riccio M., Maraldi N.M., Girbes T., Stirpe F. (2004). Interaction of volkensin with hela cells: Binding, uptake, intracellular localization, degradation and exocytosis. Cell. Mol. Life Sci..

[37] Darling R.G., Catlett C.L., Huebner K.D., Jarrett D.G. (2002). Threats in bioterrorism i: Cdc category a agents. Emerg. Med. Clin. N. Am..

[38] Bhalla D.K., Warheit D.B. (2004). Biological agents with potential for misuse: A historical perspective and defensive measures. Toxicol. Appl. Pharmacol..

[39] Senveli S.U., Tigli O. (2013). Biosensors in the small scale: Methods and technology trends. IET Nanobiotechnol..

[40] Juska V.B., Pemble M.E. (2020). A critical review of electrochemical glucose sensing: Evolution of biosensor platforms based on advanced nanosystems. Sensors.

[41] Wang J. (2001). Glucose biosensors: 40 years of advances and challenges. Electroanalysis.

[42] Yoo E.H., Lee S.Y. (2010). Glucose biosensors: An overview of use in clinical practice. Sensors.

[43] Aydin E.B., Aydin M., Sezginturk M.K. (2019). Advances in electrochemical immunosensors. Adv. Clin. Chem..

[44] Tokranova N., Cady N., Lamphere A., Levitsky I.A. (2022). Highly sensitive fentanyl detection based on nanoporous electrochemical immunosensors. IEEE Sens. J..

[45] Nunez F.A., Castro A.C.H., de Oliveira V.L., Lima A.C., Oliveira J.R., de Medeiros G.X., Sasahara G.L., Santos K.S., Lanfredi A.J.C., Alves W.A. (2023). Electrochemical immunosensors based on zinc oxide nanorods for detection of antibodies against SARS-CoV-2 spike protein in convalescent and vaccinated individuals. ACS Biomater. Sci. Eng..

[46] Zhang J., Yang L.X., Pei J., Tian Y.Z., Liu J.Y. (2022). A reagentless electrochemical immunosensor for sensitive detection of carcinoembryonic antigen based on the interface with redox probe-modified electron transfer wires and effectively immobilized antibody. Front. Chem..

[47] You Y., Luo B., Wang C., Dong H.T., Wang X.D., Hou P.C., Sun L.J., Li A.X. (2023). An ultrasensitive probe-free electrochemical immunosensor for gibberellins employing polydopamine-antibody nanoparticles modified electrode. Bioelectrochemistry.

[48] Sadique M.A., Yadav S., Khare V., Khan R., Tripathi G.K., Khare P.S. (2022). Functionalized titanium dioxide nanoparticle-based electrochemical immunosensor for detection of SARS-CoV-2 antibody. Diagnostics.

[49] Park C., Lee J., Lee D., Jang J. (2022). Paper-based electrochemical peptide sensor for label-free and rapid detection of airborne bacillus anthracis simulant spores. Sens. Actuator B-Chem..

[50] Song S.P., Wang L.H., Li J., Zhao J.L., Fan C.H. (2008). Aptamer-based biosensors. Trac-Trends Anal. Chem..

[51] Zheng J., Cheng G.F., Feng W.J., He P.G., Fang Y.Z. (2010). A thermodynamic investigation into the binding affinity between aptamer-DNA and aptamer-protein. Acta Chim. Sin..

[52] Mazzaracchio V., Neagu D., Porchetta A., Marcoccio E., Pomponi A., Faggioni G., D’Amore N., Notargiacomo A., Pea M., Moscone D. (2019). A label-free impedimetric aptasensor for the detection of bacillus anthracis spore simulant. Biosens. Bioelectron..

[53] Choi J.S., Kim S.G., Lahousse M., Park H.Y., Park H.C., Jeong B., Kim J., Kim S.K., Yoon M.Y. (2011). Screening and characterization of high-affinity ssdna aptamers against anthrax protective antigen. J. Biomol. Screen.

[54] Shao Y.L., Duan J.Q., Wang M., Cao J., She Y.X., Cao Z., Li G.Y., Jin F., Wang J., Abd El-Aty A.M. (2023). Application of molecularly imprinted electrochemical biomimetic sensors for detecting small molecule food contaminants. Polymers.

[55] Maria C.G.A., Varghese A., Nidhin M. (2023). Recent advances in nanomaterials based molecularly imprinted electrochemical sensors. Crit. Rev. Anal. Chem..

[56] Cui B.C., Liu P., Liu X.J., Liu S.Z., Zhang Z.H. (2020). Molecularly imprinted polymers for electrochemical detection and analysis: Progress and perspectives. J. Mater. Res. Technol-JMRT.

[57] Saxena K., Murti B.T., Yang P.K., Malhotra B.D., Chauhan N., Jain U. (2022). Fabrication of a molecularly imprinted nano-interface-based electrochemical biosensor for the detection of caga virulence factors of h. Pylori. Biosensors.

[58] Fan L., Zhang Q., Wang F., Yang H.F. (2023). Dummy molecularly imprinted solid-phase extraction-sers determination of afb1 in peanut. Spectroc. Acta Part A-Molec. Biomolec. Spectr..

[59] Mehmandoust M., Soylak M., Erk N. (2023). Innovative molecularly imprinted electrochemical sensor for the nanomolar detection of tenofovir as an anti-hiv drug. Talanta.

[60] Carinelli S., Kuhnemund M., Nilsson M., Pividori M.I. (2017). Yoctomole electrochemical genosensing of ebola virus cdna by rolling circle and circle to circle amplification. Biosens. Bioelectron..

[61] Ilkhani H., Farhad S. (2018). A novel electrochemical DNA biosensor for ebola virus detection. Anal. Biochem..

[62] Cooper K.L., Bandara A.B., Wang Y.M., Wang A.B., Inzana T.J. (2011). Photonic biosensor assays to detect and distinguish subspecies of francisella tularensis. Sensors.

[63] Euler M., Wang Y.J., Heidenreich D., Patel P., Strohmeier O., Hakenberg S., Niedrig M., Hufert F.T., Weidmann M. (2013). Development of a panel of recombinase polymerase amplification assays for detection of biothreat agents. J. Clin. Microbiol..

[64] Komarova E., Aldissi M., Bogomolova A. (2005). Direct electrochemical sensor for fast reagent-free DNA detection. Biosens. Bioelectron..

[65] Hao R.Z., Song H.B., Zuo G.M., Yang R.F., Wei H.P., Wang D.B., Cui Z.Q., Zhang Z.P., Cheng Z.X., Zhang X.E. (2011). DNA probe functionalized qcm biosensor based on gold nanoparticle amplification for bacillus anthracis detection. Biosens. Bioelectron..

[66] Xiao S.Y., Zhen S.J., Huang C.Z., Li Y.F. (2021). Ultrasensitive ratiometric electrochemiluminescence for detecting atxa mrna using luminol-encapsulated liposome as effectively amplified signal labels. Biosens. Bioelectron..

[67] Wasiewska L.A., Diaz F.G., Shao H., Burgess C.M., Duffy G., O’Riordan A. (2022). Highly sensitive electrochemical sensor for the detection of shiga toxin-producing e. Coli (stec) using interdigitated micro-electrodes selectively modified with a chitosan-gold nanocomposite. Electrochim. Acta.

[68] Pohanka M. (2021). COVID-19 molecular level laboratory diagnoses. Bratisl. Med. J..

[69] Pohanka M. (2021). Point-of-care diagnosis of COVID-19 disease based on antigen tests. Bratisl. Med. J..

[70] Pickering S., Betancor G., Galão R.P., Merrick B., Signell A.W., Wilson H.D., Kia Ik M.T., Seow J., Graham C., Acors S. (2020). Comparative assessment of multiple COVID-19 serological technologies supports continued evaluation of point-of-care lateral flow assays in hospital and community healthcare settings. PLoS Pathog..

[71] Ristic M., Nikolic N., Cabarkapa V., Turkulov V., Petrovic V. (2021). Validation of the standard q COVID-19 antigen test in vojvodina, serbia. PLoS ONE.

[72] Kabir M.A., Ahmed R., Iqbal S.M.A., Chowdhury R., Paulmurugan R., Demirci U., Asghar W. (2021). Diagnosis for COVID-19: Current status and future prospects. Expert Rev. Mol. Diagn..

[73] Jia Y., Sun H., Tian J.P., Song Q.M., Zhang W.W. (2021). Paper-based point-of-care testing of SARS-CoV-2. Front. Bioeng. Biotechnol..

[74] Donaldson K.A., Kramer M.F., Lim D.V. (2004). A rapid detection method for vaccinia virus, the surrogate for smallpox virus. Biosen. Bioelectron..

[75] Nath N., Eldefrawi M., Wright J., Darwin D., Huestis M. (1999). A rapid reusable fiber optic biosensor for detecting cocaine metabolites in urine. J. Anal. Toxicol..

[76] Narang U., Anderson G.P., Ligler F.S., Burans J. (1997). Fiber optic-based biosensor for ricin. Biosens. Bioelectron..

[77] Cao L.K., Anderson G.P., Ligler F.S., Ezzell J. (1995). Detection of yersinia pestis fraction 1 antigen with a fiber optic biosensor. J. Clin. Microbiol..

[78] DeMarco D.R., Saaski E.W., McCrae D.A., Lim D.V. (1999). Rapid detection of escherichia coli o157:H7 in ground beef using a fiber-optic biosensor. J. Food. Prot..

[79] Tempelman L.A., King K.D., Anderson G.P., Ligler F.S. (1996). Quantitating staphylococcal enterotoxin b in diverse media using a portable fiber-optic biosensor. Anal. Biochem..

[80] Anderson G.P., King K.D., Gaffney K.L., Johnson L.H. (2000). Multi-analyte interrogation using the fiber optic biosensor. Biosens. Bioelectron..

[81] Pohanka M. (2019). Current trends in the biosensors for biological warfare agents assay. Materials.

[82] Gwyn S., Mitchell A., Dean D., Mkocha H., Handali S., Martin D.L. (2016). Lateral flow-based antibody testing for chlamydia trachomatis. J. Immunol. Methods.

[83] Shome R., Kalleshamurthy T., Shome B.R., Sahay S., Natesan K., Bambal R.G., Sairiwal L., Mohandoss N., Barbuddhe S.B. (2018). Lateral flow assay for brucellosis testing in multiple livestock species. J. Microbiol. Methods.

[84] Machiesky L., Cote O., Kirkegaard L.H., Mefferd S.C., Larkin C. (2019). A rapid lateral flow immunoassay for identity testing of biotherapeutics. J. Immunol. Methods.

[85] Yang X.D., Wang Y.B., Yang J.F., Sun Z.K., Yue Z.H., Li L.L., He L., Hu X.F. (2019). An immunochromatographic lateral flow strip test for the rapid detection of danofloxacin in milk. Food Anal. Meth..

[86] Tel O.Y., Gurbilek S.E., Keskin O., Yucetepe A.G., Karadenizli A. (2022). Development of lateral flow test for serological diagnosis of tularemia. Kafkas Univ. Vet. Fak. Derg..

[87] Peto T., Uk C.-L.F.O. (2021). COVID-19: Rapid antigen detection for SARS-CoV-2 by lateral flow assay: A national systematic evaluation of sensitivity and specificity for mass-testing. EClinicalMedicine.

[88] Hu J.L., Xu X.X., Xu L.G., Kuang H., Xu C.L., Guo L.L. (2023). Gold nanoparticle-based lateral flow immunoassay for the rapid and on-site detection of wheat allergen in milk. Food Biosci..

[89] Slotved H.C., Sparding N., Tanassi J.T., Steenhard N.R., Heegaard N.H.H. (2014). Evaluating 6 ricin field detection assays. Biosecur. Bioterror..

[90] Gessler F., Pagel-Wieder S., Avondet M.A., Bohnel H. (2007). Evaluation of lateral flow assays for the detection of botulinum neurotoxin type a and their application in laboratory diagnosis of botulism. Diagn. Microbiol. Infect. Dis..

[91] Jia X.F., Wang K.L., Li X.Y., Liu Z.Z., Liu Y., Xiao R., Wang S.Q. (2022). Highly sensitive detection of three protein toxins via sers-lateral flow immunoassay based on sio2@au nanoparticles. Nanomed.-Nanotechnol. Biol. Med..

[92] Saxena K., Kumar A., Chauhan N., Khanuja M., Malhotra B.D., Jain U. (2023). Electrochemical immunosensor for detection of h. Pylori secretory protein vaca on g-c3n4/zno nanocomposite-modified au electrode. ACS Omega.

[93] Feng S.S., Hu W., Pei F.B., Liu Z.W., Du B., Mu X.H., Liu B., Hao Q.L., Lei W., Tong Z.Y. (2022). A highly sensitive fluorescence and screen-printed electrodes-electrochemiluminescence immunosensor for ricin detection based on cdse/zns qds with dual signal. Toxins.

[94] Atanasova M., Vasileva N., Godjevargova T. (2017). Determination of aflatoxin m1 in milk by a magnetic nanoparticle-based fluorescent immunoassay. Anal. Lett..

[95] Peltomaa R., Abbas A., Yli-Mattila T., Lamminmaki U. (2022). Single-step noncompetitive immunocomplex immunoassay for rapid aflatoxin detection. Food Chem..

[96] Cheng H.P., Chuang H.S. (2019). Rapid and sensitive nano-immunosensors for botulinum. ACS Sens..

[97] Parvin S., Hashemi P., Afkhami A., Ghanei M., Bagheri H. (2022). Simultaneous determination of bont/a and/e using an electrochemical sandwich immunoassay based on the nanomagnetic immunosensing platform. Chemosphere.

[98] Kumar D.N., Baider Z., Elad D., Blum S.E., Shtenberg G. (2021). Botulinum neurotoxin-c detection using nanostructured porous silicon interferometer. Chemosensors.

[99] Wang B., Park B., Chen J., He X.H. (2020). Rapid and label-free immunosensing of shiga toxin subtypes with surface plasmon resonance imaging. Toxins.

[100] Luo L., Yang J.W., Li Z., Xu H., Guo L., Wang L.L., Wang Y.X., Luo L.L., Wang J., Zhang P.P. (2022). Label-free differentiation and quantification of ricin, abrin from their agglutinin biotoxins by surface plasmon resonance. Talanta.

[101] Stern D., Pauly D., Zydek M., Muller C., Avondet M.A., Worbs S., Lisdat F., Dorner M.B., Dorner B.G. (2016). Simultaneous differentiation and quantification of ricin and agglutinin by an antibody-sandwich surface plasmon resonance sensor. Biosens. Bioelectron..

[102] Cagnani G.R., Oliveira T.D., Mattioli I.A., Sedenho G.C., Castro K.P.R., Crespilho F.N. (2022). From research to market: Correlation between publications, patent filings, and investments in development and production of technological innovations in biosensors. Anal. Bioanal. Chem..

[103] Lin C.T., Wang S.M. (2005). Biosensor commercialization strategy—A theoretical approach. Front. Biosci..

